# Dupless: Toward a patient‐friendly approach for erectile dysfunction nature differentiation – a study of 291 penile duplex Doppler ultrasound assessments

**DOI:** 10.1111/andr.70017

**Published:** 2025-02-27

**Authors:** Hille J. Torenvlied, Kim E. de Jager, Loes I. Segerink, Rob C. M. Pelger, Jack J. H. Beck

**Affiliations:** ^1^ Department of Urology St. Antonius Ziekenhuis Nieuwegein the Netherlands; ^2^ BIOS Lab on a Chip Group, University of Twente Enschede the Netherlands; ^3^ Department of Urology Leids Universitair Medisch Centrum Leiden the Netherlands

**Keywords:** Diagnostic screening, erectile dysfunction, erectile dysfunction etiology, patient characteristics, penile duplex doppler ultrasound

## Abstract

**Background:**

Erectile dysfunction (ED) is a condition commonly classified as either psychogenic or organic. Traditional age‐based categorizations are considered overly simplistic, yet many clinicians continue to rely on initial evaluation—patient symptoms and history, physical examination, blood tests, and questionnaires—for diagnosis due to limited modern tools.

**Objectives:**

This study aims to evaluate the predictive value of patient characteristics in individuals with “ED of indeterminate origin” following initial evaluation. Identifying these variables could enhance early diagnosis and reduce reliance on invasive procedures.

**Materials and methods:**

A retrospective cohort study was conducted on patients who underwent penile duplex Doppler ultrasound between January 2018 and January 2024 due to “ED of indeterminate origin”. Patient data, including demographics, lifestyle factors, and medical history, were collected and analyzed using unpaired *t*‐tests, chi‐squared tests, Fisher's exact tests, and multivariate logistic regression to assess their predictive value.

**Results:**

Among the 291 patients in the cohort, 165 (56.7%) were diagnosed with organic ED and 113 (38.8%) with psychogenic ED. Significant differences in age, history of diabetes mellitus, and drug use were noted. Logistic regression revealed multicollinearity among the variables and explained only 5.8% of the variance in ED etiology. Subgroup analysis revealed that diabetes mellitus predicts organic ED in patients aged 40 years and older, while psychopathology is linked to psychogenic ED. No significant predictors were identified for patients under 40 years.

**Discussion and conclusion:**

The findings of this “Dupless” study highlight the limitations of relying solely on initial evaluation to differentiate ED etiology, stressing the need for additional diagnostic tools. While some predictive factors were identified, they proved insufficient for clinical use. Thus, an urgent need exists for the development of modern, noninvasive diagnostic tools to enhance ED classification. Future research could explore machine learning models to uncover complex patterns not evident in traditional statistical methods.

## 1 INTRODUCTION

Erectile dysfunction (ED), a common condition in men, is traditionally classified as either psychogenic or organic in origin.^1^ Historically, men under 40 were primarily diagnosed with psychogenic ED, while those over 40 were presumed to have organic causes. However, cohort studies over the past decades have demonstrated that this age‐based dichotomy is overly simplistic.^2^, ^3^ In addition to age, numerous other risk factors for ED have been identified and are widely used in clinical practice.^1^, ^4^, ^5^, ^6^ Nevertheless, findings across studies exhibit significant variability and occasional contradictions regarding risk factors for ED, leading to debate over the diagnostic applicability.^7^, ^8^

Effective treatment of ED, which can significantly improve quality of life, relies on accurate diagnostic differentiation between psychogenic and organic causes.^1^ Since the 1980s, advances in diagnostic techniques have offered valuable insights into ED etiology. However, a lack of modernization has led to the discontinuation of clinical application of the diagnostic technologies.^9^ As a result, hospitals often rely on clinical evaluations—such as patient symptoms and history, physical examination, blood tests, and standardized questionnaires such as the International Index of Erectile Function (IIEF‐5) and Aging Male Symptoms (AMS) scores—to determine the origin of ED.^1^ This trend is also reflected in recent cohort studies, which differentiated between ED etiology based on initial screening.^10^, ^11^

Patients with a clearly defined ED etiology can be referred directly for treatment. All other patients with IIEF‐5 scores below 21, are classified as having “ED of indeterminate origin” and should undergo further diagnostic testing according to the European Association of Urology (EAU) guidelines.^1^ This approach acknowledges the rising prevalence of organic ED in younger men and the significant proportion (40.7%) of psychogenic ED cases in men over 40 years.^4^, ^8^

In the absence of a patient‐friendly device for nocturnal penile tumescence and rigidity (NPTR) assessment, penile duplex Doppler ultrasound (PDDU) is employed to provide diagnostic clarification. Although PDDU is a highly effective diagnostic tool, its invasiveness and high cost poses barriers to widespread use.^12^ This highlights the urgent need to “Dupless”, reducing the reliance on invasive diagnostics while ensuring accurate ED classification.

**FIGURE 1:**
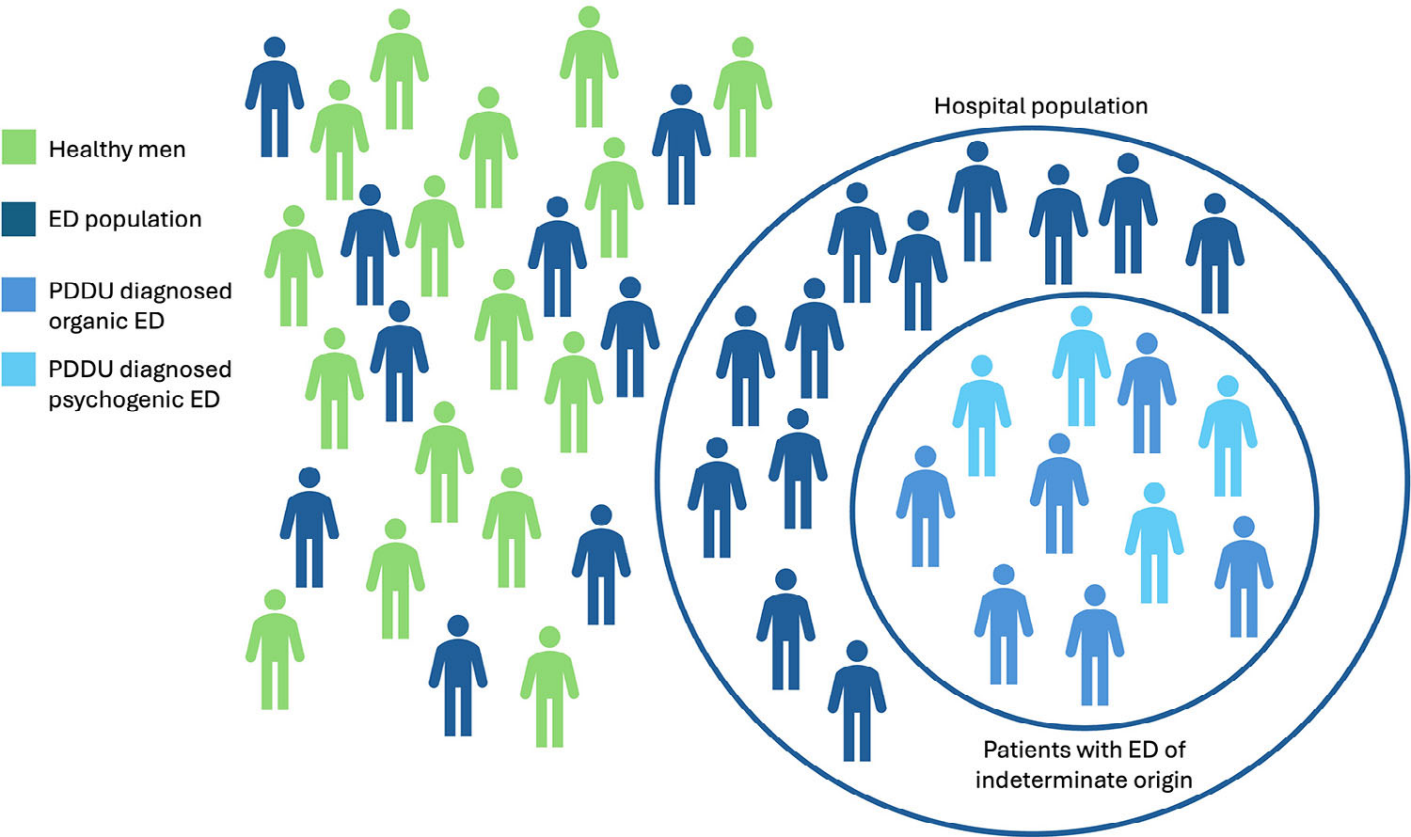
Schematic representation of the target population of patients with “erectile dysfunction (ED) of indeterminate origin” within the general population. This diagram is not to scale and does not represent actual prevalences. PDDU, penile duplex Doppler ultrasound.

To “Dupless”, the predictive value of patient characteristics in determining ED etiology in patients with ED of indeterminate origin should be investigated (see Figure 1). By identifying predictive variables, the study seeks to optimize initial screenings, potentially reducing the need for invasive procedures while maintaining accurate ED classification to improve treatment outcomes and patient's quality of life.

## 2 MATERIALS AND METHODS

At St. Antonius hospital, a specialized andrology center in the Netherlands, ED patients undergo a comprehensive initial screening based on the abovementioned evaluations (i.e., anamnesis, blood tests, and questionnaires). Patients with clearly identifiable organic causes, whether neurogenic (e.g., post‐prostatectomy) or hormonal (e.g., hypogonadism), as well as patients exhibiting signs suggestive of psychogenic ED (i.e., consistent morning erections), are promptly referred for targeted treatment. After excluding these cases for further diagnostics procedures, the remaining patients—classified as having “ED of indeterminate origin”—are subjected to PDDU for advanced assessment to rule out vascular causes of organic ED.

This retrospective cohort study was conducted at the Department of Urology at the St. Antonius hospital, Nieuwegein, the Netherlands. Ethical approval was obtained from the Medical Research Ethics Committees United on September 26, 2023 (W23.199). The study included all patients who underwent PDDU between January 2018 and January 2024 due to “ED of indeterminate origin” following initial evaluation.

PDDU was performed using a standardized protocol after intracavernosal injection of prostaglandin E1 (PGE1), with all procedures conducted by the same nurse specialist and urologist in accordance with guideline standards.^1^, ^12^ Relevant clinical data were extracted from patient records. Table 1 provides an overview of the variables considered, which were selected on the basis of their identification as key risk factors for ED in prior studies.^1^, ^6^, ^13^, ^14^, ^15^, ^16^, ^17^ In the Netherlands patients are referred to the hospital when ED etiology remains unclear after initial evaluation by the general practitioner, who specifically focusses on assessment of onset and context of ED.^18^ Thus, these characteristics are filtered at the primary care level before hospital referral.

**TABLE 1:** Overview of the variables considered in the “Dupless” study, extracted from the patient records.

**General**
ED nature
Age
BMI
IIEF‐5 score
**Lifestyle**
Smoking
Alcohol consumption
Drugs use
**Medical history**
Cardiovascular diseases, e.g., *hypertension, hypercholesterolemia, myocardial infarction, CABG, PCI, DVT*
DM *Type I & Type II DM*
Psychopathology, e.g., *burn‐out, depression, ADHD, PTSD*
Pelvic floor hypertonicity
Penile trauma
Asthma

*Note*: Related conditions were grouped to allow for dichotomization of the variables.

Medical history variables were dichotomized, and related conditions were grouped when appropriate. ED classification was determined using PPDU, with patients diagnosed with organic ED if their peak systolic velocity (PSV) was below 30 cm/s and/or their resistive index (RI) was below 0.80.^1^, ^12^, ^19^ Patients were excluded if they had an IIEF‐5 score above 21, missing medical history data, absent recording of PSV or RI values, or if they did not consent to data use.

### 2.1 Statistical analysis

In the literature, psychopathology was associated with the lowest odds ratio (OR) for ED at 1.36.^14^ A right‐tailed test for variance was conducted with a significance level of 0.05 and a power of 0.80. The chi‐squared power calculation indicated a minimum required sample size of *n* = 129.

Outcomes of continuous variables were presented as medians with standard deviations (SD), and distribution was assessed using histograms. Binary variables were reported as frequencies and proportions. Group comparisons were performed using various statistical techniques. Patients with inconclusive PDDU results were excluded from group comparisons. Continuous variables were analyzed using unpaired *t*‐tests, and *p*‐values were reported. Dichotomous variables were compared using chi‐squared tests of independence or Fisher's exact tests, the latter being used when any group had a prevalence of *n* ≤ 5. For chi‐squared tests, *p*‐values, degrees of freedom (df) and chi‐squared statistics were reported, while effect size for Fisher's Exact tests was shown as ORs. Multivariate logistic regression was employed to compare the organic and psychogenic ED groups, identify confounding factors, and assess the predictive value of the included variables.

A subanalysis was conducted using two age‐based groups, which are commonly recognized as cut‐offs for ED etiology.^20^ This subanalysis involved unpaired *t*‐tests, chi‐squared squared tests, and Fisher's exact tests. Additionally, as St. Antonius Hospital is a specialized andrology center, further analysis was conducted to assess potential referral bias in the data.^21^

Data processing was carried out using Microsoft Excel (version 16.0, Microsoft Corporation), and statistical analysis were performed with Stata (version 18, StataCorp LLC) and R Studio (version 4.3.1, Posit). The threshold for statistical significance was set at α < 0.05.

## 3 RESULTS

A total of 291 patients, with a mean age of 44.95 years (SD 14.14), met the study's inclusion criteria, accounting for 14% of all ED diagnoses in the investigated timeframe. Of these men, corresponding with 14% of the total number of patients diagnosed with ED at the St. Antonius hospital in the given timeframe, 165 patients (56.7%) were diagnosed with organic ED, with a mean PSV of 25.2 cm/s (SD 10.72) and mean RI of 0.77 (SD 0.12). These patients received intracavernosal injections of PGE1 at a mean dose of 15.13 µg (SD 5.08). The most common causes of organic ED were reduced arterial inflow (*n* = 68) and venous leakage (*n* = 64). Among the 113 patients (38.8%) diagnosed with psychogenic ED, the mean PSV was 32.7 cm/s (SD 12.5) and the mean RI was 0.88 (SD 0.57). In 13 patients (4.5%), results were inconclusive. These individuals were excluded from further analysis. PDDU examinations were performed by the urologist for 10 patients, while the remainder were examined by the nurse specialist.

To assess potential referral bias, data from the 87 patients referred from other hospitals was analyzed. In this group, 57 patients (65.5%) had organic ED, 27 (31.1%) psychogenic ED, and 3 (3.45%) inconclusive results. No significant differences in age or BMI were observed between referral and non‐referral patients. However, the IIEF‐5 score was significantly lower (*p* = 0.017) in the referral group (mean 7.80) compared to the non‐referral group (mean 10.28). Further analysis revealed no significant differences in medical history between referral and non‐referral patients with psychogenic ED. In contrast, among organic ED patients, a significantly higher proportion of those in the non‐referral group had cardiovascular disease (*p* = 0.001, *X* ^2 ^= 10.23, df = 1).

Table 2 summarizes the characteristics of patients with organic and psychogenic ED. Significant differences were observed between the two groups in age, a history of diabetes mellitus (DM) favoring organic ED, and drug use favoring psychogenic ED. Two multivariate logistic regression model were developed for these variables, with outcomes presented in Tables 3 and 4. The model in Table 3 replicates previous research showing a significant effect of DM on etiology, controlling for age.^22^ Table 4 shows that the introduction of drug use increases the overall explanatory power, but suppresses the significance of both DM and drug use due to multicollinearity; there is a significant (*p* = 0.000) correlation between drug use and age at –0.36.^23^ Despite testing several alternative models, the model of Table 4 has the highest explanatory power at 5.8%.

**TABLE 2:** Overview of the patient characteristics of the organic and psychogenic patient populations.

	Organic ED *n* = 165		Psychogenic ED *n* = 113		p‐value
	n (%)	Mean (SD)	n (%)	Mean (SD)	(df, *X* ^2^ | OR)
**Demographics**					
Age (years)	165 (100)	47.27 (14.53)	113 (100)	41.55 (13.36)	< 0.01*
BMI (kg/m^2^)	114 (69.1)	26.65 (4.18)	71 (62.8)	26.23 (3.57)	0.24
IIEF‐5 score	65 (39.4)	8.82 (5.24)	44 (38.9)	10.75 (5.64)	0.07
**Lifestyle**					
*Smoking*					0.12
Never	84 (50.9)		45 (39.8)		(3, 4.21)
Former	32 (19.4)		32 (28.3)		
Current	32 (19.4)		24 (21.2)		
Unknown	17 (10.3)		12 (10.6)		
*Alcohol*					0.42
No	35 (21.2)		29 (25.7)		(2, 0.65)
Yes	107 (64.8)		70 (61.9)		
Unknown	23 (13.9)		13 (11.5)		
*Drugs*					0.007*
Never	117 (70.9)		68 (60.2)		(3, 9.82)
Former	5 (3.0)		9 (8.0)		
Current	10 (6.1)		17 (15.0)		
Unknown	33 (20.0)		19 (16.8)		
**Medical history**					
Cardiovascular diseases	42 (25.5)		19 (16.8)		0.09 (1, 2.92)
DM	19 (11.5)		2 (1.8)		0.006* (7.22)
Psychopathology	18 (10.9)		19 (16.8)		0.16 (1, 2.03)
Pelvic floor hypertonicity	11 (6.7)		8 (7.1)		0.89 (1, 0.02)
Penile trauma	8 (4.8)		3 (2.7)		0.28 (1.87)
Asthma	11 (6.7)		9 (8.0)		0.68 (1, 0.17)

*Note*: For the unpaired *t*‐tests, *p*‐values are stated. Regarding the chi‐squared (X^2^)‐tests, additionally the degrees of freedom (df) and X^2^‐statistics are provided. For Fisher's Exact tests, the effect size is shown through the OR. Statistically significant outcomes (p < 0.05) are marked with *.

**TABLE 3:** Outcomes of the multivariate logistic regression model for age and diabetes mellitus (DM) (*n* = 278).

Predictor	β (Coefficient)	OR	95% CI for OR	*p*‐value
Age	−0.03	0.97	0.96–0.99	0.002*
DM	−1.46	0.23	0.07–0.82	0.023*

*Note*: Statistically significant outcomes (p < 0.05) are marked with *.

**TABLE 4:** Outcomes of the multivariate logistic regression model for the patient characteristics showing significant differences between the organic and psychogenic erectile dysfunction (ED) groups (*n* = 226).

Predictor	β (Coefficient)	OR	95% CI for OR	*p*‐value
Age	−0.02	0.98	0.96–1.00	0.029*
Drugs usage	0.39	1.48	0.95–2.31	0.084
DM	−1.14	0.32	0.09–1.16	0.083

*Note*: Statistically significant outcomes (p < 0.05) are marked with *.

**FIGURE 2:**
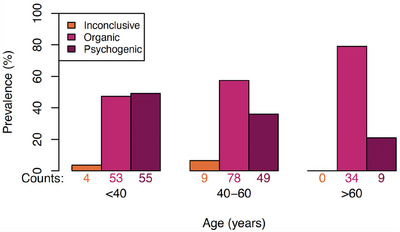
Distribution of prevalences of erectile dysfunction (ED) etiology diagnoses by age group.

Figure 2 shows the distribution of ED diagnoses by age, highlighting the relationship between age and ED etiology. Notably, in patients under 40 years, there was a near‐equal distribution of organic and psychogenic ED cases, suggesting that age alone is an unreliable predictor. Further statistical analysis comparing patients aged < 40 years and ≥ 40 years revealed significant differences in BMI (*p* = 0.003), drugs use (*p* < < 0.001, *X^2 ^* = 30.93, df = 3), cardiovascular disease (*p* < < 0.001, *X^2^* = 19.10, df = 1), DM (*p* = 0.029, OR = 0.32), and penile trauma (*p *= 0.022, OR = 4.45).

In patients aged ≥ 40 years, those with organic ED were significantly older (*p* = 0.037, mean age 55.58) than those with psychogenic ED (mean age 52.72). No significant differences were observed in BMI or IIEF‐5 scores within either age group. Table 5 presents the age‐based subgroup analysis results, showing that in patients aged ≥ 40 years, organic ED is significantly associated with DM (*p* = 0.022), while psychogenic ED is linked to psychopathology (*p* = 0.030). A multivariate logistic regression model was developed for these variables, with results showing that the pseudo‐R‐squared is 4.1% and thus minimally explains the variance in ED etiology for this subpopulation. Regarding the patients aged < 40 years, no significant differences were identified between organic and psychogenic ED.

**TABLE 5:** Outcomes of the bivariate statistical age‐related subgroup analysis.

	< 40 years *n* = 108			≥ 40 years *n* = 170		
	Organic ED (*n* = 53)	Psychogenic ED (*n* = 55)	p‐value (df, X^2^ | OR)	Organic ED (*n* = 112)	Psychogenic ED (*n* = 58)	p‐value (df, X^2^ | OR)
Number of patients, *n* (%)	n (%)	n (%)		n (%)	n (%)	
**Lifestyle**						
*Smoking*			0.14			0.62
Never	24 (45.3)	19 (34.5)	(3, 3.88)	60 (53.6)	26 (44.8)	(3, 0.97)
Former	7 (13.2)	16 (29.1)		25 (22.3)	16 (27.6)	
Current	12 (22.6)	14 (25.5)		20 (17.9)	10 (17.2)	
Unknown	10 (18.9)	6 (10.9)		7 (6.3)	6 (10.3)	
*Alcohol*			0.91			0.14
No	8 (15.1)	10 (18.2)	(2, 0.013)	27 (24.1)	19 (32.8)	(2, 2.14)
Yes	34 (64.2)	40 (72.7)		73 (65.2)	30 (51.7)	
Unknown	11 (20.8)	5 (9.1)		12 (10.7)	9 (15.5)	
*Drugs*			0.089			0.41
Never	31 (58.5)	27 (49.1)	(3, 4.84)	86 (76.8)	4 (85.4)	(3, 1.79)
Former	2 (3.8)	8 (14.5)		3 (2.7)	1 (1.7)	
Current	8 (15.1)	14 (25.5)		2 (1.8)	3 (5.2)	
Unknown	12 (22.6)	6 (10.9)		21 (18.8)	12 (22.4)	
**Medical history**						
Cardiovascular diseases	5 (9.4)	4 (7.3)	0.48 (1.33)	37 (33.0)	15 (25.9)	0.34 (1, 0.93)
DM	3 (5.7)	1 (1.8)	0.30 (3.24)	16 (14.3)	2 (3.4)	0.022* (4.67)
Psychopathology	8 (15.1)	7 (12.7)	0.72 (1, 0.13)	10 (8.9)	12 (20.7)	0.030* (1, 4.69)
Pelvic floor hypertonicity	4 (7.5)	7 (12.7)	0.29 (0.56)	7 (6.3)	1 (1.7)	0.18 (0.26)
Penile trauma	5 (9.4)	3 (5.5)	0.34 (1.81)	3 (2.7)	0 (0)	0.28 (‐)
Asthma	4 (7.5)	4 (7.3)	0.62 (1.04)	7 (6.3%)	5 (8.6%)	0.39 (0.71)

*Note*: For the unpaired *t*‐tests, *p*‐values are stated. Regarding the *X^2^*‐tests, additionally the degrees of freedom (df) and X^2^‐statistics are provided. For Fisher's Exact tests, the effect size is shown through the OR. Statistically significant outcomes (p < 0.05) are marked with *.

### 3.1 Discussion and Conclusion

The objective of this study was to identify predictive patient characteristics in cases of “ED of indeterminate origin” after initial screening. Optimizing initial screening would lead to “Dupless” by reducing the need for additional diagnostic procedures and could potentially result in improved efficiency in clinical practice. In this cohort study of 291 patients undergoing PDDU, various analyses were conducted to differentiate between organic and psychogenic ED. However, the predictive value of the patient's characteristics was minimal and insufficient for reliable clinical application. This suggests that further refining initial screening based on the patient characteristics alone is not a feasible approach for ED differentiation. Consequently, accurate ED classification—and thus optimized treatment outcomes—remains reliant on additional diagnostic procedures.

This is the first study to examine the predictive value of patient characteristics for ED etiology in a cohort undergoing PDDU following inconclusive initial evaluation. The study included data of 291 patients, surpassing the minimum sample size (*n* = 129) calculated in the power analysis, ensuring robust analysis. Furthermore, corrections were made for statistical accuracy for the variables with low prevalence. Significant differences between organic and psychogenic ED groups were identified for age, IIEF‐5 scores, history of DM, and drug use, aligning with existing literature on ED risk factors.^8^, ^13^ However, logistic regression analysis revealed multicollinearity with age, diminishing the predictive value of these characteristics.^23^ In the final regression model, only 5.8% of the variance in ED etiology could be explained.

To address the issue of multicollinearity, age‐based subgroup analyses were performed. In patients aged ≥ 40 years, a history of DM showed predictive value for organic ED, while psychopathology significantly favored psychogenic ED. However, the clinical relevance of these findings is questionable. While 16 out of 18 patients with DM would be correctly diagnosed with organic ED, 2 would receive incorrect treatment. More notably, considering all patients aged ≥ 40 years with psychopathology (*n* = 22) as having psychogenic ED would result in 10 misdiagnoses, raising ethical concerns about acceptable levels of diagnostic error in initial screening. The absence of studies evaluating the sensitivity and specificity of current ED diagnosis based solely on initial screening further complicates this issue. Nevertheless, early identification of organic ED is critical, not only for improving quality of life but also for preventing potentially life‐threatening conditions and reducing long‐term healthcare costs.^1^, ^24^ This underscores the importance of performance of additional diagnostic procedures when ED etiology is unclear.^3^

In patients aged < 40 years, no predictive value was found for patient characteristics in distinguishing organic and psychogenic ED. Interestingly, despite the historical tendency to diagnose younger patients with psychogenic ED, the case distribution in this cohort was almost equal – 53 cases of organic ED and 55 of psychogenic ED. This finding aligns with previous studies that indicate a notable prevalence of organic ED among younger patients.^23^

A common complication in psychogenic ED is the development of a vicious cycle, where psychological factors exacerbate the condition. Cognitive distractions often linked to a loss of confidence and heightened stress responses during sexual activity, further impair arousal.^25^ Similarly, organic ED can lead to diminished confidence, which in turn contributes to the development of multifactorial ED, where both organic and psychological factors are intertwined.^3^ For instance, among patients in this study, three individuals diagnosed with psychogenic ED following penile trauma reported stress from past pain and anxiety about erectile function, further impacting their condition. The multifactorial nature of ED, particularly in younger patients, complicates its differentiation based on initial screening. The overlap of organic and psychogenic presentation of symptoms underscores the limitations of relying solely on initial screening for diagnosis and reinforces the need for additional, more nuanced diagnostic techniques.

### 3.2 Limitations

Of the 291 patients included in this study, 13 had inconclusive PDDU results, primarily due to elevated stress levels during the examination. The invasive nature of PDDU can increase stress and affect diagnostic accuracy. Elevated adrenaline levels can induce smooth muscle contraction, leading to false‐positive diagnoses of organic ED due to impaired veno‐occlusive function, mimicking venous leakage.^26^ Further research into long‐term treatment outcomes is needed to better understand this effect in the “Dupless” cohort. Another potential source of false‐positive diagnoses of organic ED could stem from PGE1 underdosing. However, the variations in PGE1 dosing among the patients suggest that assessors adjusted doses to ensure sufficient pharmacologic response for accurate evaluation. Although overdiagnosis of organic ED cannot be completely ruled out, the homogeneity of the cohort supports the validity of the findings.

Notably, 97% of PDDU assessments in this study were performed by the same nurse specialist. As PDDU is operator‐specific, potential bias can be introduced with this approach. However, ensuring procedural consistency is critical for accurate interpretation of PDDU results as highlighted by Nascimento et al.^26^ In this study, this consistency was ensured by having almost all PDDU assessments performed by a single operator. To rule out the potential bias, it would be valuable to replicate the study in a different clinical setting, involving multiple operators, to compare outcomes and further evaluate the generalizability of the findings.

This study is the first to investigate a large cohort of patients undergoing additional diagnostic screening for “ED of indeterminate origin”. Conducted at the St. Antonius Hospital, a specialized andrology center in the Netherlands, the cohort included 87 patients referred from other centers nationwide. Statistical analysis revealed two significant differences between referral and non‐referral patients: the referral group had a lower IIEF‐5 score and a lower prevalence of cardiovascular disease. The lower IIEF‐5 score in the referral group likely reflects the fact that these patients were referred when primary care could not adequately manage the severity of condition. For patients with cardiovascular disease, differences in diagnostic criteria between centers may indicate that they were diagnosed with organic ED during initial screening. Since all other variables were comparable between the two groups, the likelihood of referral bias significantly impacting the study outcomes is minimal.

### 3.3 Recommendations

This study demonstrated the absence of predictive value for patient characteristics in differentiating ED etiology using traditional statistical methods. However, developing a machine learning model based on the data from this cohort could potentially uncover predictive relationships that were not evident in this study. At present, the results of this study suggest that it is not possible to reduce the need for additional diagnostic procedures based solely on patient characteristics. Without modernized tools for non‐invasive diagnostic assessment, clinicians will have to continue to rely on the invasive PDDU for further assessment. Thus, there is an urgent need within the field of andrology for the development of a modern, non‐invasive diagnostic tool to better differentiate ED etiology.

## AUTHOR CONTRIBUTIONS

Hille J. Torenvlied and Kim E. de Jager performed the research, including the research design, data acquisition, and analysis. Hille J. Torenvlied conducted the interpretation of the data and drafted the paper. Loes I. Segerink, Rob C. M. Pelger, and Jack J. H. Beck contributed to the research design, interpretation of the data, and have revised the manuscript. All authors approve the submitted version of this article.

## FUNDING INFORMATION

This research was not funded by any sponsor or public, commercial, or not‐for‐profit funder.

## CONFLICT OF INTEREST STATEMENT

The authors declare no conflicts of interest.
